# Skin Tenting Due to Displaced Mid-shaft Clavicle Fracture in an Adolescent

**DOI:** 10.7759/cureus.88772

**Published:** 2025-07-25

**Authors:** Lillian Toaspern, Morteza Khodaee

**Affiliations:** 1 Family Medicine, University of Colorado School of Medicine, Denver, USA; 2 Sports Medicine, University of Colorado, Denver, USA

**Keywords:** comminuted fracture, neurovascular complication, skiing, sport, trauma

## Abstract

Sport-related clavicle fractures are a relatively common injury in the pediatric population. However, neurovascular complications at initial presentation are rare in adolescents. We report the case of a 14-year-old female skier who presented with a left shoulder injury and visible skin tenting. Plain radiographs demonstrated a comminuted and displaced mid-shaft clavicle fracture. Due to concern for impending necrosis and potential conversion to an open fracture, she was promptly referred to a nearby facility with surgical capabilities. She underwent surgical fixation shortly after arrival, with a favorable postoperative course and full recovery. This case highlights the importance of early recognition of potential neurovascular compromise in mid-shaft clavicle fractures and underscores the need for timely referral and definitive management.

## Introduction

Winter sports such as skiing and snowboarding are growing in popularity, with over 200 million participants worldwide [1]. In the United States alone, approximately 10 million individuals actively participate in these activities [2]. Children and adolescents represent nearly one-third of these athletes and account for a similar proportion of skiing- and snowboarding-related injuries [1-3]. Among this population, fractures are the most common type of injury, with the shoulder being the second most frequently affected anatomical region [3]. The clavicle is the most commonly fractured bone in the shoulder girdle in children, accounting for 10%-15% of all pediatric fractures [4]. Approximately half of these injuries are sports-related [5]. In one study conducted at a ski resort, nearly one-third of clavicle fractures occurred in individuals under the age of 18 [6]. The mid-shaft is the most common anatomical location for clavicle fractures [6,7]. Neurovascular complications can be present at the time of injury, may develop over time, or may result from iatrogenic causes [8-10]. Neurovascular complications may arise as a result of the fracture itself or its management, particularly surgical intervention [5-10]. These complications include brachial plexus injury and injuries to the subclavian vessels, such as pseudoaneurysm formation [6-10]. Given the high incidence of clavicular injuries and the high-risk mechanisms associated with winter sports, effective management of acute mid-shaft clavicle fractures is a critical skill for clinicians treating this population. Here, we present a case of skin tenting secondary to a displaced mid-shaft clavicle fracture in an adolescent. Although this presentation is uncommon among adolescents, prompt recognition and appropriate management are critical.

## Case presentation

**Figure 1:**
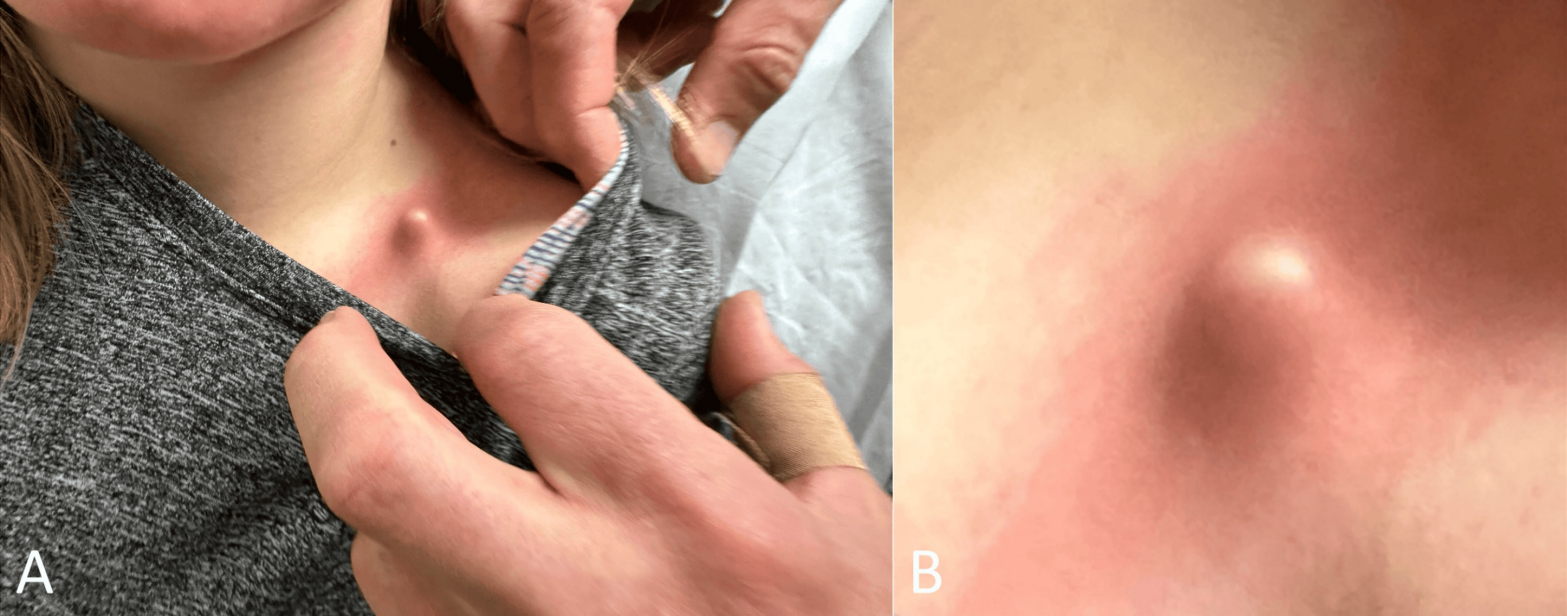
Photographs of the left shoulder on arrival showing a blanched protuberant mid-clavicle deformity (A and B)

A 14-year-old girl presented to the Denver Health Winter Park Medical Center shortly after falling on her left shoulder while racing in a ski tournament. This community medical facility and level V trauma center is located at the base of the resort, approximately 90 minutes from Denver, Colorado. She was accompanied by her father, who provided consent for the publication of this case without any identifiable information. She felt an immediate pain in her left mid-clavicle region. She described the pain as mild without moving her left arm. Her past medical and surgical histories were otherwise unremarkable. On physical examination, there was a blanched and bulged left mid-clavicle deformity (Figure 1).

**Figure 2:**
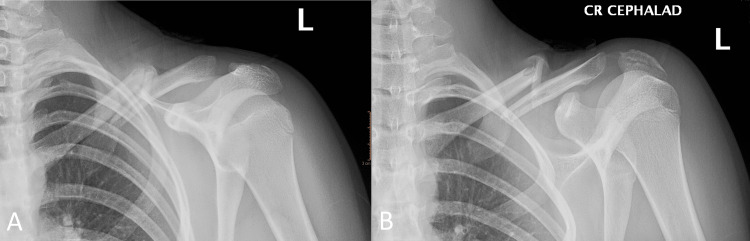
Left clavicle plain radiographs: anteroposterior and cephalad views demonstrate a comminuted and inferiorly displaced (lateral section) mid-shaft clavicular fracture with an approximate 3 shafts width of bayonet opposition and an approximate 1 ½ shaft width of inferior displacement (A and B)

Otherwise, she was neurovascularly intact. Plain radiography demonstrated a comminuted and displaced mid-shaft left clavicle fracture (Figure 2). She was transferred to a facility (an hour away) with surgical capability for an urgent surgery. Clavicle fixation surgery was performed on the same day with no complications and good postoperative recovery.

## Discussion

There has been ongoing debate regarding the indications for surgical intervention in displaced mid-shaft clavicle fractures in adolescents [11-13]. Currently, the universally accepted indications for operative management in pediatric patients include open fractures, acute neurovascular compromise, floating shoulder, and significant skin tenting with concern for impending necrosis [4,7,11-13]. Among these, skin tenting presents a unique diagnostic challenge due to its subjective nature and considerable interobserver variability [13-16].

This variability arises, in part, from the absence of standardized guidelines for physical examination findings related to skin tenting [13-16]. Different providers may apply the term inconsistently; some referring to an angular prominence of the skin overlying the fracture, while others interpret it as blanching or thinning of the skin, suggestive of compromised perfusion [13-16]. Given that skin tenting may represent an impending open fracture, timely and accurate diagnosis is critical [14].

Despite its clinical significance, skin tenting is relatively uncommon, and epidemiological data, particularly in pediatric populations, are limited. Three small-scale studies report its incidence ranging from 4% to 9% in adults [16,17] and up to 12% in adolescents [13]. The risk factors associated with skin tenting include medial-lateral fracture shortening and lower body mass index (BMI), while it appears to be unrelated to fracture comminution or superior-inferior displacement [13,16].

Although surgical fixation has traditionally been favored in the management of displaced mid-shaft clavicle fractures, recent evidence suggests that nonoperative treatment may be appropriate in carefully selected adolescent patients [7,11-14,16-19]. Nevertheless, when skin tenting poses a risk to the integrity of the overlying skin, raising the possibility of necrosis or progression to an open fracture, operative intervention is warranted [13,14,16]. To better guide clinical decision-making, further large-scale prospective research is necessary to clarify surgical indications and to develop standardized criteria for the assessment and management of skin tenting.

## Conclusions

Skin tenting is an uncommon complication of mid-shaft clavicular fractures, but it can lead to neurovascular compromise and skin breakdown. Therefore, accurate diagnosis is essential to ensure appropriate management. Although no universally accepted standard protocol exists, the benefits and risks of each treatment strategy should be evaluated on a case‑by‑case basis. While surgical fixation has traditionally been standard, recent studies indicate that nonoperative approaches may be appropriate in select cases. However, when skin tenting threatens the integrity of the overlying tissue, raising concerns for necrosis or conversion to an open fracture, prompt reduction or surgical intervention is indicated.
